# Self-amplifying nicotinamide N-methyltransferase feedback loops: Drivers of anticancer drug resistance and therapeutic opportunities

**DOI:** 10.1016/j.jpha.2026.101588

**Published:** 2026-02-16

**Authors:** Shuang-Zhou Bi, Xiang-Li Ni, Wei-Dong Sun, Chen-Ying Zhang, An Liu, Shi-Yan Lai, Jiang-Hua Li

**Affiliations:** Physical Education College, Jiangxi Normal University, Nanchang, 330022, China

**Keywords:** Nicotinamide N-methyltransferase, Drug resistance, Feedback loops, Lactate metabolism, Therapeutic targeting

## Abstract

Drug resistance poses a significant challenge in cancer therapy, undermining treatment efficacy and patient survival. Nicotinamide N-methyltransferase (NNMT), a pivotal cytoplasmic enzyme, plays a crucial role in mediating drug resistance by linking nicotinamide adenine dinucleotide (oxidized form) (NAD^+^) metabolism and the S-adenosylmethionine/S-adenosylhomocysteine (SAM/SAH) methylation cycle. Notably, self-amplifying NNMT feedback loops in cancer cells drive the development of resistance to anticancer drugs. NNMT reduces the cytotoxicity of chemotherapeutic agents, drives resistance to targeted therapies such as kinase inhibitors and monoclonal antibodies, and facilitates immune evasion, thereby diminishing the efficacy of immunotherapy. It also amplifies resistance through self-reinforcing feedback loops, including the NNMT/aldehyde dehydrogenase 3 family member A1 (ALDH3A1)/lactate/NNMT and early growth response 1 (EGR1)/NNMT/EGR1 circuits, which intensify resistance signaling within the tumor microenvironment. While novel therapeutic strategies targeting NNMT, such as gene silencing, small-molecule inhibitors, and feedback circuit interventions, show promise in reversing resistance, their clinical translation remains challenging. Future research should focus on elucidating the interplay between NNMT and lactate metabolism (including exercise-modulated lactylation), identifying additional NNMT-associated feedback loops, and investigating NNMT's regulatory roles in alternative metabolic pathways. These efforts may yield new insights and therapeutic targets to overcome drug resistance and improve clinical outcomes for cancer patients.

## Highlights

• NNMT promotes drug resistance via multiple mechanisms.
• NNMT's self-activating feedback loops amplify resistance signals.
• NNMT significantly increases the risk of cancer cell drug resistance.
• High NNMT levels diminish anticancer drug sensitivity and treatment efficacy.
• NNMT-targeted therapies restore anticancer drug sensitivity in preclinical models.

## Graphical abstract

**Figure:**
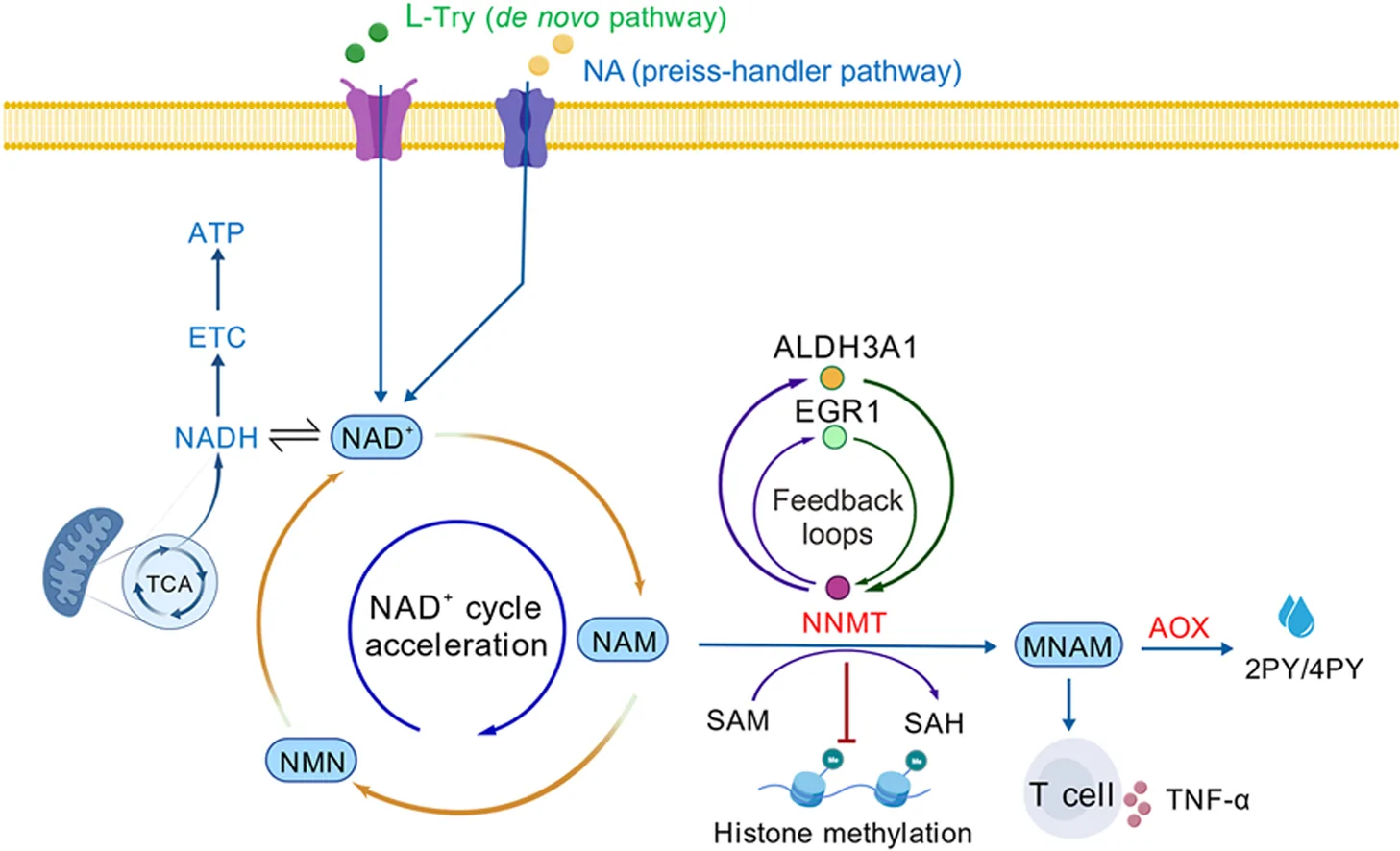

## 1 Introduction

Resistance to anticancer drugs represents one of the most formidable challenges in oncology, severely limiting the durability of therapeutic responses and patient outcomes [[1], [2], [3]]. For instance, in advanced non-small cell lung cancer (NSCLC), while epidermal growth factor receptor-tyrosine kinase inhibitors (EGFR-TKIs) provide initial clinical benefits for *EGFR*-mutated cases, drug resistance ultimately develops in nearly all patients, leading to tumor relapse and metastatic progression [1]. Despite recent advancements in immunotherapy and molecular targeted therapies, drug resistance remains pervasive across various malignancies and is characterized by considerable heterogeneity [4,5]. Thus, unraveling the molecular underpinnings of drug resistance and identifying novel therapeutic targets are essential to enhancing patient benefit.

Nicotinamide N-methyltransferase (NNMT) is an enzyme that catalyzes the methylation of nicotinamide (NAM) to form 1-methylnicotinamide (MNAM), utilizing S-adenosylmethionine (SAM) as a methyl donor and generating S-adenosylhomocysteine (SAH) as a byproduct. Through this reaction, NNMT connects intracellular nicotinamide adenine dinucleotide (oxidized form) (NAD^+^) metabolism with the SAM/SAH methylation cycle [[6], [7], [8]]. NNMT is highly expressed in both adipose tissue and the liver. In contrast, its expression is significantly lower throughout the rest of the body [9]. Previous studies have demonstrated that NNMT is overexpressed in various cancers [[10], [11], [12], [13], [14]] and plays a significant role in tumorigenesis and progression through mechanisms involving epigenetics, energy metabolism, and increased MNAM levels [15]. As these mechanisms have been extensively discussed in existing literature reviews [[16], [17], [18], [19], [20]], this review therefore focuses on the specific role of NNMT in cancer drug resistance to provide a new perspective on this clinical challenge.

An increasing body of research has implicated NNMT in cancer drug resistance. For example, Hsu et al. [21] analyzed the correlation between cancer-associated genes and the sensitivity of 99 antitumor drugs, revealing that NNMT expression may influence chemotherapeutic responsiveness. Similarly, Yu et al. [22] demonstrated that silencing NNMT reduced cellular resistance to rapamycin, whereas NNMT overexpression conferred enhanced resistance. In xenograft models, Cui et al. [23] found that NNMT knockdown suppressed tumor growth and increased sensitivity to 5-fluorouracil (5-FU). Wang et al. [24] reported that NNMT promoted resistance to paclitaxel (PTX) and doxorubicin (ADM) in breast cancer cells by enhancing the activity of sirtuin 1 (SIRT1). As demonstrated by Heide et al. [25], NNMT serves as a pivotal regulator of cancer-associated fibroblasts (CAFs) and represents a therapeutic target for alleviating immune suppression within the tumor microenvironment. Another study by Wang et al. [26] further demonstrated that NNMT upregulation contributes to EGFR-TKI resistance through SIRT1-mediated deacetylation of proto-oncogene c-myc (c-Myc), which promotes glycolytic reprogramming. In bladder cancer, elevated NNMT expression has been linked to radiotherapy resistance [27]. Wang et al. [28] also identified NNMT as a driver of tumor progression and 5-FU resistance in gastric cancer. Notably, recent research in small-cell lung cancer revealed the presence of self-sustaining NNMT feedback loops, specifically NNMT/aldehyde dehydrogenase 3 family member A1 (ALDH3A1)/lactate/NNMT and early growth response 1 (EGR1)/NNMT/EGR1, that synergistically promote resistance to EGFR-TKIs [29]. As a metabolic-epigenetic integrator, NNMT remodels the tumor microenvironment and sustains drug resistance via positive feedback loops, establishing it as a prime target for mechanistic and therapeutic exploration.

## 2 Multifaceted mechanisms of NNMT-mediated resistance to anticancer drugs

According to the classification by the U.S. National Cancer Institute (NCI), the primary drug-based treatment strategies for cancer currently include chemotherapy, targeted therapy, and immunotherapy. The major drugs used in these three therapies, as well as the mechanisms of NNMT-mediated resistance to them, have been summarized in Table 1 [[23], [24], [25], [26], [27], [28], [29], [30], [31], [32], [33], [34], [35], [36], [37], [38], [39], [40], [41], [42], [43]]. In the following sections, these mechanisms will be discussed separately.

**Table 1:** Major drugs of the primary drug-based treatment strategies for cancer and corresponding nicotinamide N-methyltransferase (NNMT)-associated resistance mechanisms.

Therapy strategy	Drug category	Representative drugs	Core NNMT-mediated resistance mechanisms	Key molecular pathways	Cancer type(s)	Refs.
Chemotherapy	Cytotoxic drugs	5-FU, PTX, and ADM	Glycolytic reprogramming (enhanced Warburg effect), apoptosis suppression, and ABC transporter-mediated drug efflux	SIRT1/c-Myc/*HK2-LDHA*, PI3K/Akt/p53-ASK, and MRP2/ABCB1	ESCC, NSCLC, CRC, GC, and BC	[23,24,28,[31], [32], [33], [34], [35]]
Targeted therapy	Small-molecule kinase inhibitors	EGFR-TKIs and BRAF inhibitors	Epigenetic remodeling (H3K9me3/H3K27me3↓) and metabolic-signaling crosstalk	EGR1/NNMT positive feedback loop and ALDH3A1-lactate-H3K18la	BC, LC, NSCLC, and MM	[26,27,29,[36], [37], [38]]
	Monoclonal antibodies	Anti-VEGF (bevacizumab)	Microenvironment acidification promoting invasion and alternative energy utilization (ketone bodies/α-methylgalactoside)	ZEB1/NNMT/EMT axis	SOC, HCC, and GBM	[[39], [40], [41]]
	Proteostasis-targeting drugs	Proteasome inhibitors (bortezomib)	Compensatory stress pathway activation (Hedgehog activation)	SIRT1-GLI2-Hedgehog pathway	MM	[42]
	Metabolic inhibitors (OXPHOS inhibitors)	Gboxin, oligomycin A, IACS010759, and berberine	NNMT-DNMT1-regulated OXPHOS dependency	NNMT-DNMT1 axis	CRA	[43]
Immunotherapy	ICIs	Anti-PD-1 and anti-PD-L1	CAF-mediated immunosuppressive microenvironment, macrophage M2 polarization	NNMT^+^ CAFs activate SAA-TLR2/TGF-β axis and induce immunosuppression (via C5aR^+^ MDSCs)	BC and HGSOC	[25,30]
	Adoptive cell therapy	CAR-T cells	Not currently associated with NNMT mechanisms	−	−	−

−: no data; 5-FU: 5-fluorouracil; PTX: paclitaxel; ADM: doxorubicin; ABC: adenosine triphosphate (ATP)-binding cassette; ESCC: esophageal squamous cell carcinoma; NSCLC: non-small cell lung cancer; CRC: colorectal cancer; GC: gastric cancer; BC: breast cancer; LC: lung cancer; SIRT1: sirtuin 1; c-Myc: proto-oncogene c-myc; HK2: hexokinase 2; LDHA: lactate dehydrogenase a; PI3K/Akt: phosphoinositide 3-kinase/protein kinase B; ASK: apoptosis signal-regulating kinase; MRP2: multidrug resistance-associated protein 2; EGFR-TKIs: epidermal growth factor receptor-tyrosine kinase inhibitors; BRAF: v-raf murine sarcoma viral oncogene homolog b; EGR1: early growth response 1; ALDH3A1: aldehyde dehydrogenase 3 family member a1; H3K18la: histone h3 lysine 18 lactylation; H3K9me3: histone h3 lysine 9 trimethylation; H3K27me3: histone h3 lysine 27 trimethylation; anti-VEGF: anti-vascular endothelial growth factor; ZEB1: zinc finger e-box binding homeobox 1; EMT: epithelial-mesenchymal transition; SOC: serous ovarian cancer; HCC: hepatocellular carcinoma; GBM: glioblastoma; GLI2: glioma-associated oncogene homolog 2; OXPHOS: oxidative phosphorylation; DNMT1: DNA methyltransferase 1; CRA: colorectal adenocarcinoma; MM: multiple myeloma; ICIs: immune checkpoint inhibitors; anti-PD-1: anti-programmed death-1; anti-PD-L1: anti-programmed death-ligand 1; CAFs: cancer-associated fibroblasts; SAA: serum amyloid a; TLR2: Toll-like receptor 2; TGF-β: transforming growth factor beta; MDSCs: myeloid-derived suppressor cells; C5aR^+^: complement component 5a receptor positive; HGSOC: high-grade serous ovarian cancer; CAR-T: chimeric antigen receptor T-cells.

### 2.1 NNMT-mediated resistance to chemotherapy drugs

Chemotherapy remains one of the cornerstone treatment modalities for a wide range of cancers. Most chemotherapeutic agents function primarily by inducing cytotoxic stress through mechanisms such as DNA damage, inhibition of mitosis, or disruption of nucleotide metabolism [44,45]. According to Table 1, commonly used cytotoxic drugs include 5-FU, PTX, and ADM, which exert their anticancer effects by interfering with cell proliferation and promoting apoptosis. As illustrated in Fig. 1, NNMT reduces the cytotoxic effects of chemotherapy by enhancing anaerobic metabolism, inhibiting apoptosis, and promoting drug efflux.

**Fig. 1:**
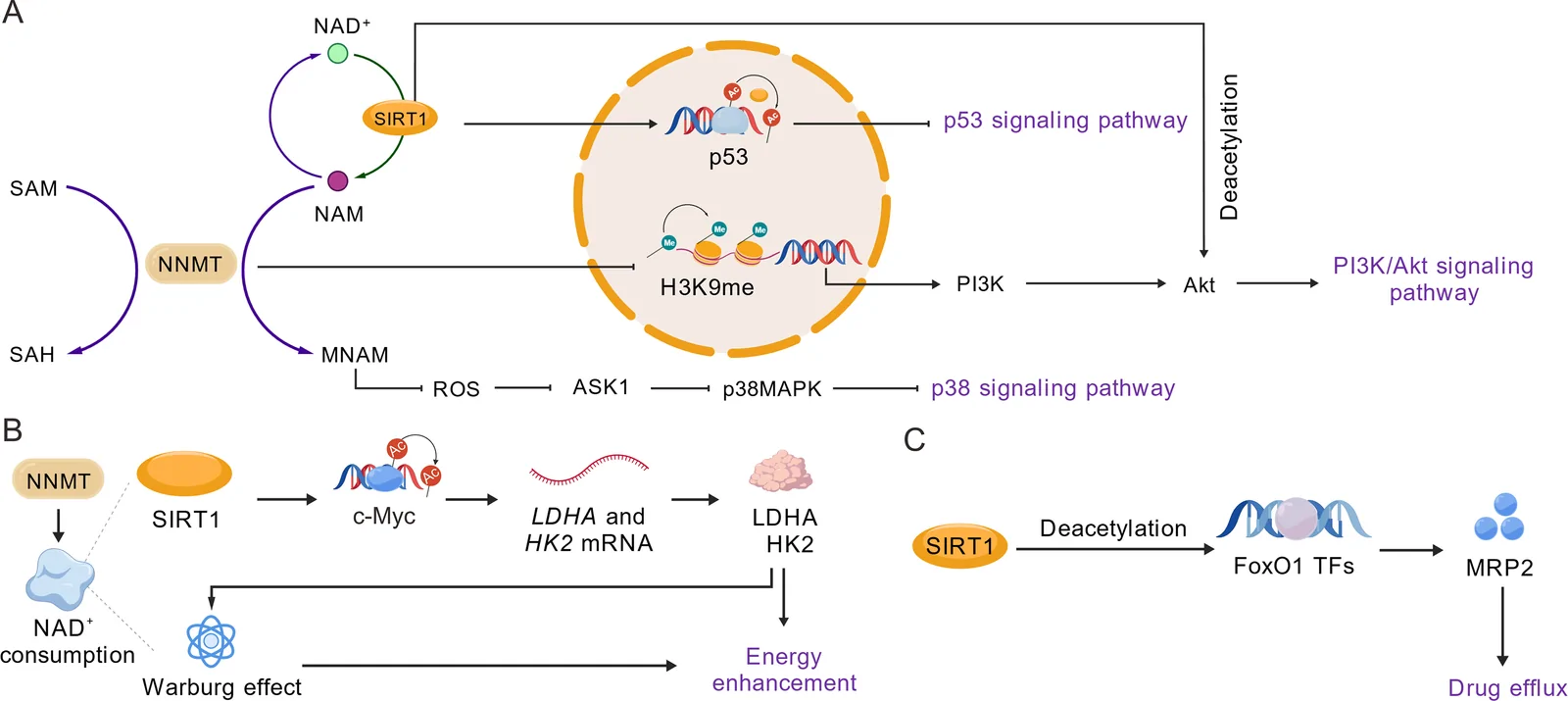
Nicotinamide N-methyltransferase (NNMT) promotes drug resistance by reducing cytotoxicity. (A) Apoptosis inhibition. (B) Enhanced anaerobic metabolism. (C) Promoted drug efflux. SAM: S-adenosylmethionine; SAH: S-adenosylhomocysteine; NAD^+^: nicotinamide adenine dinucleotide (oxidized form); NAM: nicotinamide; H3K9me: histone H3 lysine 9 methylation; PI3K: phosphoinositol-3 kinase; Akt: protein kinase B; MNAM: 1-methylnicotinamide; ROS: reactive oxygen species; ASK1: apoptosis signal-regulating kinase 1; p38MAPK: p38 mitogen-activated protein kinase; SIRT1: sirtuin 1; Ac: acetylation; c-Myc: proto-oncogene c-myc; LDHA: lactate dehydrogenase A; HK2: hexokinase 2; FoxO1 TFs: forkhead box O1 transcription factors; MRP2: multidrug resistance (MDR)-associated protein 2.

#### 2.1.1 Enhancing glycolytic reprogramming in cancer cells

Metabolic reprogramming is widely recognized as a hallmark of malignant transformation [46]. Cancer cells often adopt a glycolytic phenotype, even under normoxic conditions, to maximize ATP production, a phenomenon known as the Warburg effect [47,48]. Cui et al. [23] reported that NNMT facilitates this metabolic shift by promoting the Warburg effect, thereby reducing esophageal squamous cell carcinoma (ESCC) cells' sensitivity to 5-FU. Specifically, enhanced glycolytic flux allows tumor cells to better adapt to nutrient deprivation and oxidative stress, ultimately supporting survival and evading 5-FU-mediated cytotoxicity [23]. However, the underlying molecular mechanisms by which NNMT regulates this effect remain incompletely understood.

Based on computational simulations and mathematical modeling, Bockwoldt et al. [49] found that although NNMT reduces steady-state levels of NAD^+^, it simultaneously accelerates NAD^+^ turnover by promoting its resynthesis and enhancing NAM consumption. Since the Warburg effect requires elevated NAD^+^ availability to sustain rapid ATP generation and provide metabolic intermediates for cell proliferation [50], NNMT may support this glycolytic phenotype by increasing the overall NAD^+^ flux.

NNMT has also been shown to promote anaerobic glycolysis by modulating the activity of key glycolytic enzymes. These enzymes, such as phosphoglycerate mutase 1 (PGAM1), lactate dehydrogenase A (LDHA), and hexokinase 2 (HK2), play central roles in both tumorigenesis and therapeutic resistance [47,51]. Cui et al. [23] demonstrated that NNMT enhances glycolytic reprogramming by upregulating these enzymes, thereby reducing 5-FU sensitivity in ESCC cells.

Moreover, NNMT activation prevents NAM accumulation and alleviates feedback inhibition of NAD^+^-dependent enzymes such as SIRT1 and poly(adenosine diphosphate (ADP)-ribose) polymerase 1 (PARP1), which are known to influence resistance phenotypes [16]. Yang et al. [32] further showed that SIRT1 enhances HK2 activity through deacetylation, thereby augmenting glycolytic flux, improving antioxidant capacity, and increasing intracellular ATP levels, factors associated with cisplatin resistance in ESCC cells.

NNMT regulates glycolysis through combined transcriptional and epigenetic mechanisms. Wang et al. [26] found that elevated NNMT expression triggers SIRT1-dependent deacetylation and consequent activation of the oncogenic transcription factor c-Myc, which transcriptionally upregulates the glycolytic enzymes HK2 and LDHA. Extending these findings, Wu et al. [43] demonstrated that NNMT modulates DNA methyltransferase 1 (DNMT1) activity, which in turn affects the expression of glycolysis-related genes. Nevertheless, the direct link between these regulatory networks and cytotoxic drug sensitivity remains to be definitively established.

#### 2.1.2 Suppresses apoptotic signaling to promote chemoresistance

The tumor suppressor protein p53 plays a central role in mediating cellular responses to chemotherapy. Under normal acetylation conditions, p53 efficiently responds to DNA damage induced by anticancer drugs by activating downstream pathways that promote cell cycle arrest or apoptosis, thereby increasing drug sensitivity [52]. Wang et al. [24] reported that NNMT suppresses this apoptotic response by stabilizing SIRT1 and enhancing its deacetylase activity. This suppresses p53-mediated apoptosis, leading to reduced efficacy of chemotherapeutic agents, and ultimately contributing to drug resistance [24].

The apoptosis signal-regulating kinase 1 (ASK1)-p38 mitogen-activated protein kinase (MAPK) cascade, another critical apoptotic signaling axis, is regulated in part by p53 and is involved in coordinating the balance between autophagy and apoptosis during responses to 5-FU treatment [53]. Xie et al. [33] showed that NNMT-mediated elevation of MNAM in colorectal cancer (CRC) cells reduces 5-FU-induced reactive oxygen species (ROS) production, leading to inactivation of the ASK1-p38 MAPK pathway and suppression of apoptosis. These findings suggest a synergistic effect between the ROS-ASK1-p38 MAPK axis and the SIRT1-p53 pathway in enhancing chemoresistance.

Emerging evidence underscores NAD^+^ metabolism's pivotal role in regulating programmed cell death pathways. Niu et al. [34] demonstrated that oxaliplatin treatment activates PARP1, causing substantial NAD^+^ depletion, which in turn impairs SIRT1 activity. As a result, SIRT1's ability to regulate stress responses and apoptotic signaling is diminished, promoting oxaliplatin resistance in CRC cells. This underscores the critical role of the PARP1-NAD^+^-SIRT1 axis in chemotherapy resistance and reveals a potential link between metabolic stress and epigenetic regulation.

The phosphoinositide 3-kinase/protein kinase B (PI3K/Akt) signaling pathway is frequently activated in cancer and is known to inhibit apoptosis, thus facilitating the development of resistance [54]. In CRC, elevated ROS levels have been shown to trigger PI3K/Akt activation, upregulate hypoxia-inducible factor 1-alpha (HIF-1α), and reduce cellular sensitivity to 5-FU [55]. NNMT-induced resistance has also been linked to aberrant PI3K/Akt signaling. Wang et al. [28] found that NNMT activates this pathway by suppressing histone H3 lysine 9 (H3K9) methylation, thereby promoting both 5-FU resistance and tumor progression in gastric cancer. Targeting NNMT was shown to restore chemosensitivity, suggesting a potential therapeutic strategy to overcome drug resistance.

Phosphatase and tensin homolog (PTEN), a well-established tumor suppressor, antagonizes PI3K/Akt signaling and inhibits tumor progression in multiple cancer types [56,57]. Whether NNMT modulates PI3K/Akt signaling by regulating PTEN expression or activity remains an open question and warrants further investigation.

Furthermore, the PI3K/Akt pathway may be mediated by SIRT1 in response to NNMT's regulatory effects. Wei et al. [35] demonstrated that SIRT1 overexpression activates Akt signaling, leading to reduced apoptosis and enhanced cell proliferation. Conversely, nicotinamide treatment downregulates SIRT1, decreases its deacetylation of Akt, and subsequently sensitizes breast cancer cells to ADM [35]. Collectively, these studies suggest that NNMT promotes multidrug resistance (MDR) by activating the PI3K/Akt axis through its influence on NAD^+^ metabolism and epigenetic remodeling. Future research integrating metabolomics and single-cell transcriptomic approaches may help clarify how the NNMT-PI3K/Akt axis dynamically regulates chemoresistance in diverse tumor microenvironments.

#### 2.1.3 Facilitation of drug efflux via adenosine triphosphate (ATP)-binding cassette (ABC) transporters

MDR is a phenomenon wherein cancer cells simultaneously develop resistance to multiple chemotherapeutic agents with distinct structures and mechanisms of action [58]. One of the major contributors to MDR is the overexpression of ABC transporter proteins, particularly MDR-associated proteins (MRPs), in various tumor types [59,60]. These efflux pumps actively transport chemotherapeutic agents like PTX and ADM out of cancer cells, thereby reducing intracellular drug accumulation, diminishing cytotoxic effects, and ultimately contributing to treatment failure [61].

Recent studies have revealed a connection between NNMT and the regulation of drug efflux via epigenetic and metabolic pathways. Choi et al. [31] demonstrated that deacetylation of Forkhead box protein O1 (FoxO1) by SIRT1 is associated with the upregulation of MRP2 in tamoxifen-resistant MCF-7 breast cancer cells. Notably, inhibition of SIRT1 significantly restored drug sensitivity, highlighting the potential of the SIRT1/FoxO1 signaling axis as a therapeutic target to overcome chemoresistance.

Given that NNMT enhances SIRT1 activity by modulating NAD^+^ availability, it is plausible that NNMT indirectly facilitates drug efflux by sustaining the SIRT1/FoxO1-mediated expression of efflux transporters. This mechanism may represent a critical link between metabolic reprogramming and MDR, further underscoring the role of NNMT as a driver of chemoresistance.

### 2.2 NNMT-mediated resistance to targeted therapy drugs

Targeted therapies have transformed the landscape of cancer treatment by specifically interfering with molecular abnormalities driving tumor progression. Based on their mechanisms and molecular targets, targeted agents can be broadly classified into several categories, including small-molecule kinase inhibitors (e.g., EGFR-TKIs), monoclonal antibodies (e.g., anti-VEGF), proteostasis-targeting drugs (e.g., bortezomib), and metabolic inhibitors (e.g., metformin). These agents function by blocking oncogenic signaling pathways, disrupting protein homeostasis, or impairing cancer-specific metabolic dependencies. NNMT contributes to the resistance against these targeted agents through distinct mechanisms, which include epigenetic remodeling, metabolic reprogramming, tumor microenvironment acidification, and altered energy substrate utilization.

#### 2.2.1 Resistance to small-molecule kinase inhibitors

Activating mutations in the *EGFR* are a major driver of NSCLC progression. Therapeutic outcomes in *EGFR*-mutated NSCLC are significantly improved with early-generation EGFR-TKIs, such as erlotinib and gefitinib [62]. However, acquired resistance to EGFR-TKIs remains a major clinical challenge. Bach et al. [38] identified NNMT as a key mediator of EGFR-TKI resistance in *EGFR*-mutated NSCLC cells and demonstrated that NNMT knockdown partially reverses this drug resistance. Hou et al. [37] have demonstrated that the overexpression of NNMT enhances the resistance of lung cancer cells to AZD9291. Conversely, the inhibition of NNMT significantly improves the efficacy of the drug, particularly in resistant cells. Despite these findings, the precise molecular mechanisms through which NNMT drives acquired resistance to EGFR-TKIs remain incompletely understood.

Multiple studies have indicated that *EGFR*-mutant NSCLC cells exhibit increased dependency on glucose metabolism, and enhanced glucose uptake has been implicated as a driver of resistance to EGFR-TKIs [63,64]. As illustrated in Fig. 2, Wang et al. [26] demonstrated that elevated NNMT expression triggers SIRT1-dependent deacetylation and consequent activation of the oncogenic transcription factor c-Myc, which transcriptionally upregulates the glycolytic enzymes HK2 and LDHA. This metabolic reprogramming contributes to EGFR-TKI resistance by enhancing glucose metabolism and indirectly sustaining EGFR signaling, thereby diminishing the efficacy of kinase inhibition.

**Fig. 2:**
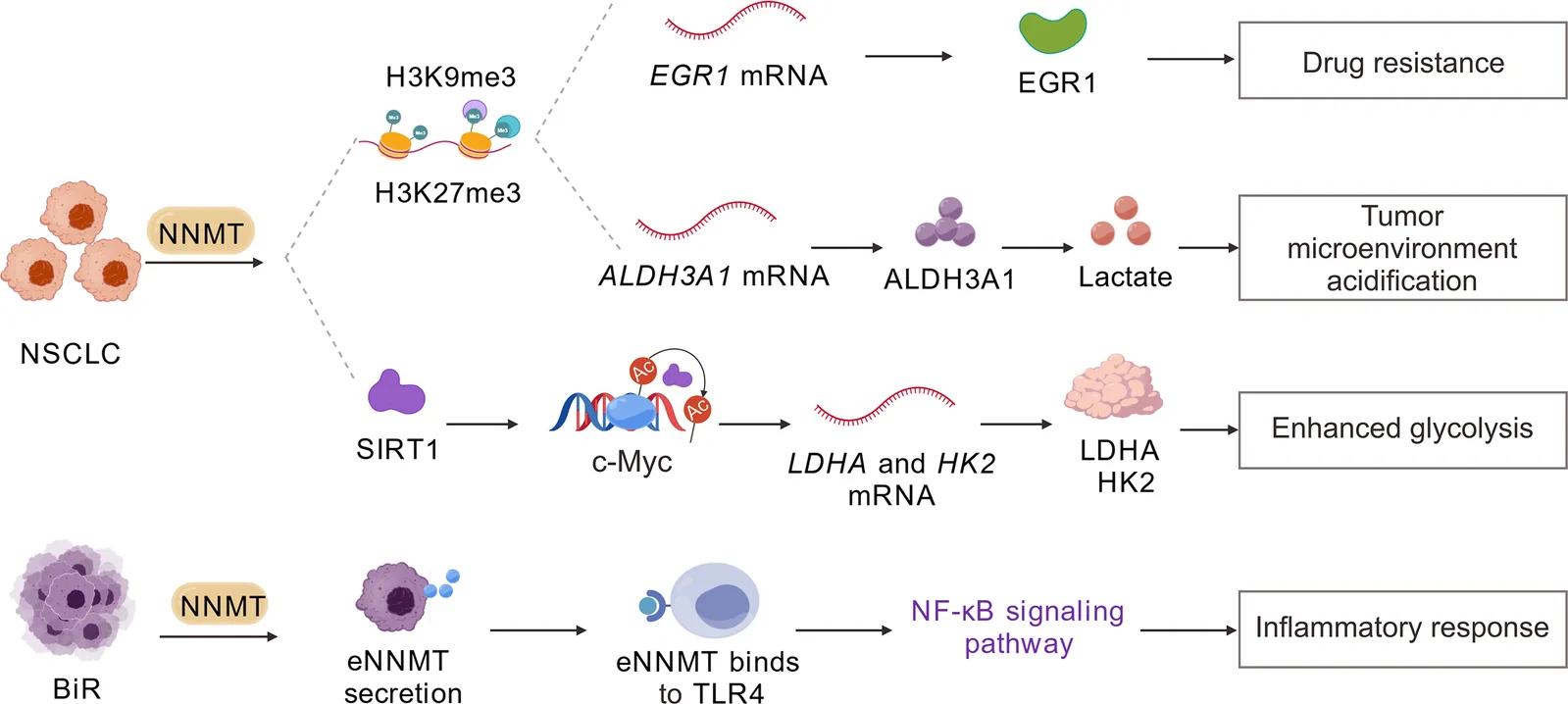
Nicotinamide N-methyltransferase (NNMT) promotes drug resistance by weakening targeted inhibition. NSCLC: non-small cell lung cancer; H3K9me3: histone H3 lysine 9 trimethylation; H3K27me3: histone H3 lysine 27 trimethylation; *EGR1* mRNA: early growth response 1 messenger RNA; *ALDH3A1* mRNA: aldehyde dehydrogenase 3 family member A1 messenger RNA; SIRT1: sirtuin 1; Ac: acetylation; c-Myc: proto-oncogene c-myc; LDHA: lactate dehydrogenase A; HK2: hexokinase 2; BiR: v-raf murine sarcoma viral oncogene homolog B (BRAF) inhibitor-resistant cells; eNNMT: extracellular NNMT; TLR4: Toll-like receptor 4; NF-κB: nuclear factor kappaB.

Clinical and preclinical evidence from Dai et al. [29] consistently showed that both acquired EGFR-TKI-resistant NSCLC cell lines and matched patient-derived tumor specimens exhibited upregulated NNMT expression. Mechanistically, NNMT depletes intracellular SAM, leading to reduced histone H3 lysine 9 and lysine 27 trimethylation (H3K9me3 and H3K27me3), thereby epigenetically activating the transcription of ALDH3A1 and EGR1. The upregulation of ALDH3A1 increases intracellular lactate levels, which not only enhances NNMT expression via p300-mediated histone H3 lysine 18 lactylation (H3K18la), but also acts as a resistance signal by altering the tumor microenvironment [29].

EGR1, a transcription factor known to regulate both tumor progression and drug resistance, has been implicated in multiple malignancies [[65], [66], [67]]. For instance, EGR1-driven metabolic reprogramming has been shown to confer ibrutinib resistance in B-cell lymphomas [67]. Collectively, these findings suggest that NNMT promotes EGFR-TKI resistance through a complex network involving epigenetic remodeling, metabolic rewiring, and tumor microenvironment acidification, with EGR1 and lactate serving as key amplifiers of the resistance phenotype.

In addition, v-raf murine sarcoma viral oncogene homolog B (BRAF) inhibitors, such as dabrafenib and vemurafenib, are classified as small-molecule kinase inhibitors. These agents specifically target the kinase activity of the BRAF protein, thereby inhibiting the overactivation of the rat sarcoma virus/rapidly accelerated fibrosarcoma/MAPK kinase/extracellular signal-regulated kinase (RAS/RAF/MEK/ERK) signaling pathway. This mechanism ultimately leads to the suppression of tumor cell proliferation and survival [68]. Ghezzi et al. [36] reported that in BRAF inhibitor-resistant (BiR) cells, the messenger RNA (mRNA) and protein expression levels of NNMT are significantly elevated. Notably, this overexpression is not confined to the intracellular environment but is also released extracellularly via extracellular vesicles (EVs) and soluble factors. The inhibition of NNMT has been shown to markedly reduce cell proliferation and enhance sensitivity to BRAF inhibitors [36]. These findings suggest that NNMT may represent a potential therapeutic target for overcoming resistance to BRAF inhibitors.

#### 2.2.2 Resistance to monoclonal antibodies

Bevacizumab, a humanized monoclonal antibody targeting VEGF, has been approved for the treatment of various solid tumors, including recurrent glioblastoma [69]. However, its clinical efficacy in glioblastoma is limited, particularly due to acquired resistance and increased tumor invasiveness following treatment failure [[70], [71], [72]]. As illustrated in Fig. 3, Kanska et al. [40] demonstrated that chronic glucose deprivation, induced by anti-angiogenic therapy such as bevacizumab, triggers upregulation of NNMT via the transcription factor ZEB1, thereby endowing tumor cells with enhanced metabolic plasticity. Mechanistically, NNMT facilitates adaptation to glucose-restricted environments by remodeling the epigenetic landscape through methylation metabolism. This enables cancer cells to exploit alternative energy substrates such as ketone bodies and α-methyl-D-galactoside, thereby reducing their reliance on angiogenesis for nutrient supply. In parallel, NNMT promotes morphological changes and reduces cell-cell adhesion, increasing the propensity for invasion and migration [40]. These phenotypic shifts are likely mediated by epigenetic alterations, as elevated NNMT expression has been associated with global changes in DNA and histone methylation patterns, which in turn influence genes involved in cell motility and metastasis [73]. Notably, Li et al. [41] provided direct evidence that NNMT overexpression reduces H3K27me3 and concurrently decreases N^6^-methyladenosine (m^6^A) modification of *cluster of differentiation 44* (*CD44*) mRNA. This promotes the expression of CD44 variant isoforms v6 and v3 on the cancer cell surface, facilitating metastatic spread. Piao et al. [39] further showed that epithelial-to-mesenchymal transition (EMT) is strongly associated with acquired resistance to bevacizumab, in which NNMT acts as a molecular hallmark of the mesenchymal tumor subtype.

**Fig. 3:**
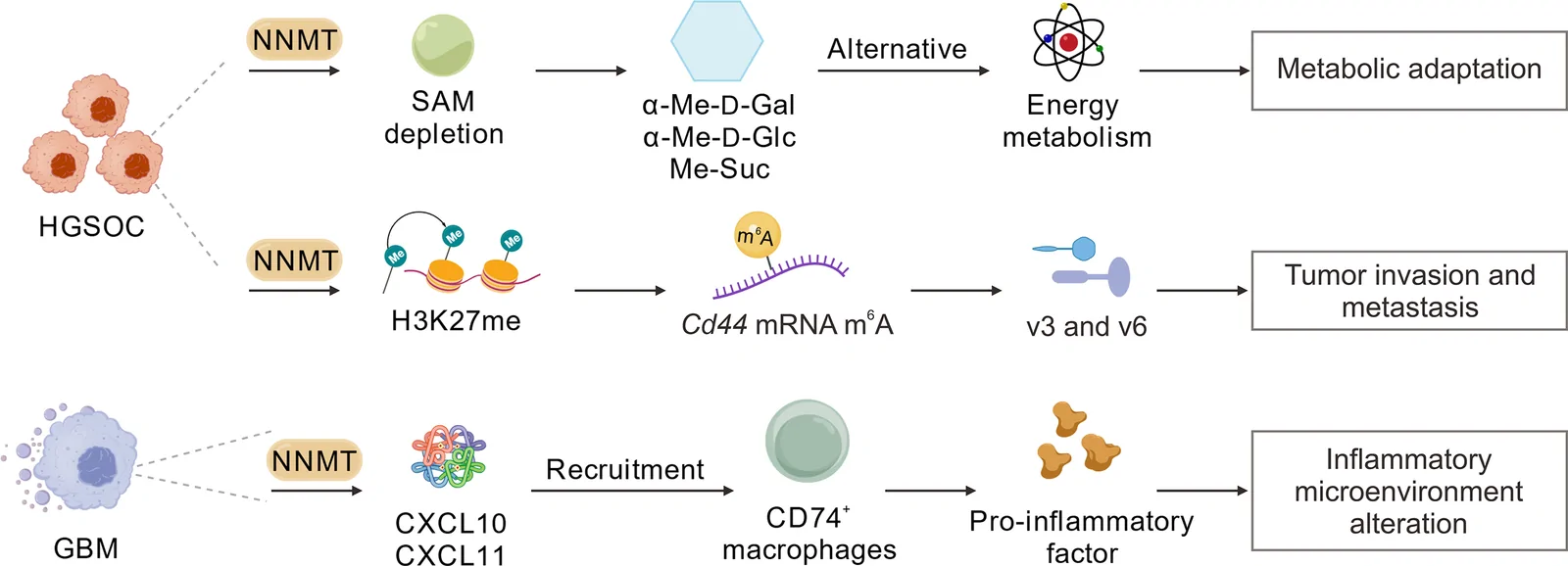
Nicotinamide N-methyltransferase (NNMT) promotes drug resistance by rendering monoclonal antibodies ineffective. HGSOC: high-grade serous ovarian carcinoma; SAM: S-adenosylmethionine; α-Me-D-Gal: alpha-methyl-D-galactose; α-Me-D-Glc: alpha-methyl-D-glucopyranose; Me-Suc: methylsuccinic acid; H3K27me: histone H3 lysine 27 methylation; CD44: cluster of differentiation 44; mRNA: messenger RNA; m^6^A: N^6^-methyladenosine; GBM: glioblastoma multiforme; CXCL10: C−X−C motif chemokine 10.

In addition to its metabolic and epigenetic roles, NNMT contributes to therapy resistance by modulating the tumor microenvironment. Specifically, NNMT upregulation enhances the expression of inflammatory chemokines such as C−X−C motif chemokine ligand 10 (CXCL10) and CXCL11, which recruit CD74^+^ tumor-associated macrophages and promote secretion of transforming growth factor beta (TGF-β), thereby establishing a pro-inflammatory and immunosuppressive milieu that favors tumor survival and resistance [39,74].

In summary, NNMT plays a multifaceted role in bevacizumab resistance by enabling metabolic adaptation through methylation-mediated reprogramming, which allows cancer cells to survive under nutrient-deprived conditions. It also alters cell-cell interactions by modifying cellular morphology and reducing adhesion, thereby enhancing invasiveness. Furthermore, NNMT contributes to remodeling the tumor microenvironment by recruiting immune cells and maintaining a pro-inflammatory state that facilitates resistance. These mechanistic insights highlight NNMT as a promising therapeutic target for combination strategies aimed at overcoming monoclonal antibody resistance.

#### 2.2.3 Resistance to proteostasis-targeting drugs

Proteasome inhibitors, such as bortezomib, exert anticancer effects by blocking proteasomal activity, thereby disrupting protein degradation and promoting intracellular accumulation of misfolded or damaged proteins, ultimately inducing apoptosis in cancer cells [75]. Although proteasome inhibitors have shown clinical efficacy, the development of drug resistance and disease relapse remains a significant therapeutic challenge [76].

As illustrated in Fig. 4, NNMT may contribute to bortezomib resistance by enhancing the activity of SIRT1. Xie et al. [42] demonstrated that SIRT1 deacetylates glioma-associated oncogene homolog (GLI) family zinc finger 2 (GLI2) at lysine 757, leading to its stabilization by reducing polyubiquitination and subsequent proteasomal degradation. As a result, intracellular accumulation of GLI2 increases, activating the Hedgehog (Hh) signaling pathway and upregulating downstream targets such as *cyclin D1* (*CCND1*) and *proto-oncogene MYC* (*MYC*) [42]. These genes are closely associated with enhanced cell survival, proliferation, and chemoresistance, effectively counteracting the pro-apoptotic effects of proteasome inhibition [77].

**Fig. 4:**
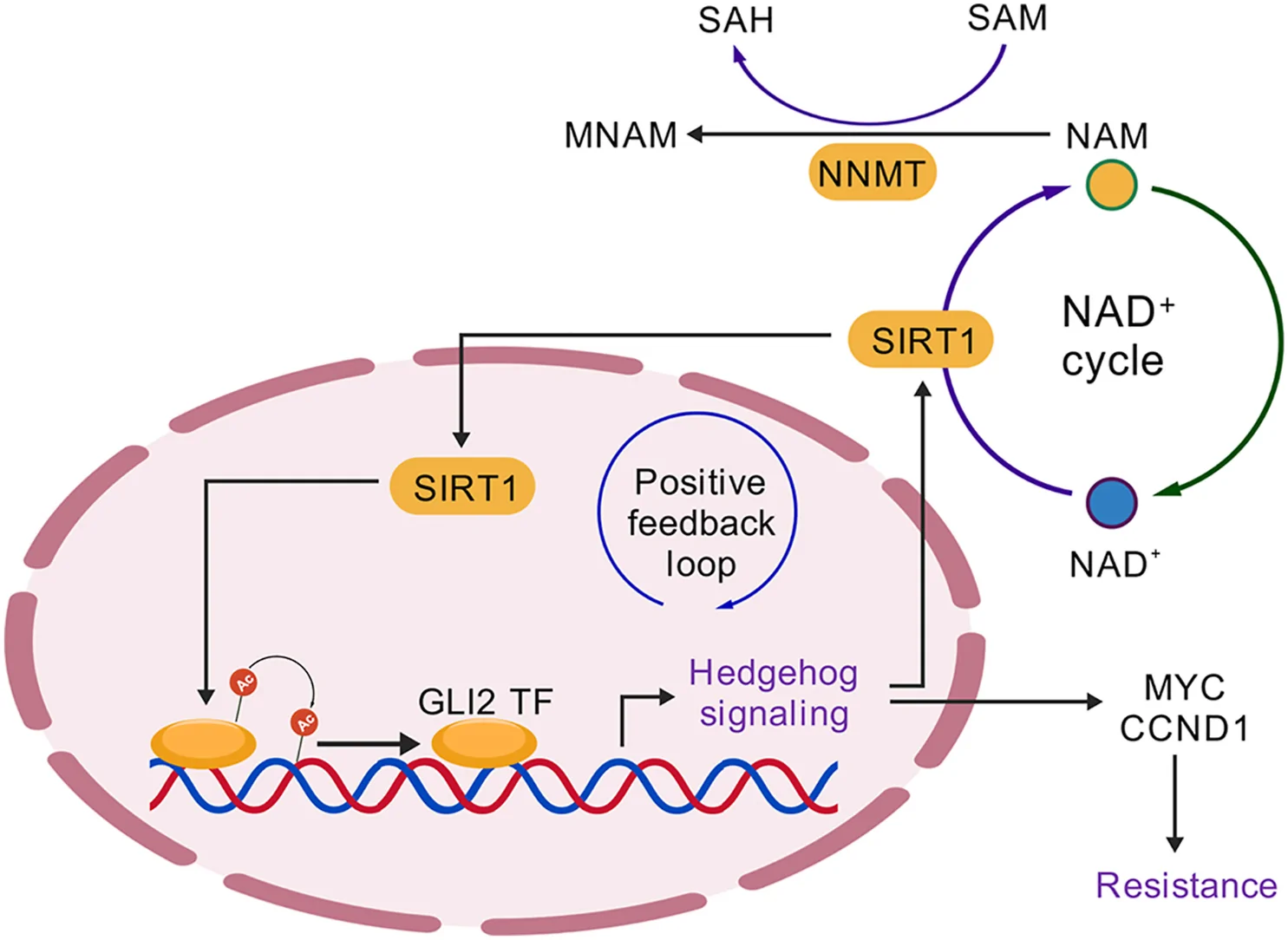
Sirtuin 1 (SIRT1) activation promotes bortezomib resistance via Hedgehog (Hh) signaling enhancement. NAM: nicotinamide; SAM: S-adenosylmethionine; SAH: S-adenosylhomocysteine; NNMT: nicotinamide N-methyltransferase; MNAM: 1-methylnicotinamide; NAD^+^: nicotinamide adenine dinucleotide (oxidized form); Ac: acetylation; GLI2: glioma-associated oncogene 2; TF: transcription factor; MYC: myelocytomatosis oncogene; Ac: acetylation; CCND1: cyclin D1.

Furthermore, SIRT1 itself has been identified as a direct transcriptional target of the Hh pathway, forming a positive feedback loop that further amplifies resistance [42]. Pharmacological inhibition of SIRT1 has been shown to partially suppress Hh pathway activation and sensitize cells to bortezomib both *in vitro* and *in vivo* [42].

Taken together, although both SIRT1 and proteasome inhibitors influence protein degradation, they operate through distinct molecular mechanisms. SIRT1-mediated deacetylation of GLI2 enhances Hh signaling, promoting cancer cell survival and resistance to proteasome inhibitors. Combining SIRT1 inhibitors with proteasome-targeting agents offers a promising strategy to overcome resistance in multiple myeloma and potentially other malignancies.

#### 2.2.4 Resistance to metabolic inhibitors

Oxidative phosphorylation (OXPHOS) inhibitors function primarily by targeting key components of the mitochondrial electron transport chain, thereby disrupting mitochondrial function and impairing energy metabolism in cancer cells [78,79]. Although these agents show therapeutic promise, there remains a lack of definitive molecular markers to predict the sensitivity of cancer cells to OXPHOS inhibition [80].

Wu et al. [43] reported that cancer cells exhibiting high sensitivity to OXPHOS inhibitors tend to display elevated baseline OXPHOS activity coupled with low expression levels of NNMT. This low NNMT expression is frequently accompanied by upregulation of DNMT1, suggesting a coordinated regulatory mechanism that maintains the cells' vulnerability to OXPHOS disruption. Interestingly, while both NNMT and DNMT1 expression levels are associated with high expression of OXPHOS-related genes, these mitochondrial genes do not appear to be direct transcriptional targets of DNMTs.

This observation implies that NNMT and DNMT1 may regulate OXPHOS gene expression through indirect or epigenetically mediated mechanisms, potentially involving broader chromatin remodeling or metabolic feedback loops. Further elucidation of these mechanisms is essential for identifying biomarkers of OXPHOS inhibitor sensitivity and for designing rational combination therapies to overcome metabolic resistance.

### 2.3 NNMT-mediated resistance to immunotherapy drugs

Immunotherapy has emerged as a revolutionary strategy in cancer treatment, aiming to harness the body's immune system to recognize and eliminate tumor cells [81]. Major immunotherapies include checkpoint inhibitors that reactivate T cells, and chimeric antigen receptor T-cell (CAR-T) cell therapy, which uses engineered immune cells to target tumors (Table 1). NNMT has been implicated in immunotherapy resistance primarily through the remodeling of the tumor microenvironment.

#### 2.3.1 CAFs with high NNMT drive resistance to immune checkpoint inhibitor*s* (ICIs)

ICIs, including anti-programmed death-1 (anti-PD-1) and anti-programmed death-ligand 1 (anti-PD-L1) antibodies, function by blocking inhibitory signals that suppress T cell activity within the tumor microenvironment. By disrupting PD-1/PD-L1 interactions, these agents restore cytotoxic T lymphocyte function and enhance antitumor immune responses, thereby providing durable clinical benefits across various malignancies [82]. However, the efficacy of ICIs in many cancers remains limited, due to complex drug resistance mechanisms that are not yet fully understood [83].

CAFs with high NNMT expression are closely associated with the formation of an immunosuppressive microenvironment. As illustrated in Fig. 5, Heide et al. [25] demonstrated that NNMT reduces histone H3K27me3 methylation by depleting SAM, leading to the upregulation of multiple immunosuppressive factors in CAFs. This process promotes the recruitment of myeloid-derived suppressor cells (MDSCs) and suppresses CD8^+^ T cell activity, thereby diminishing the efficacy of ICIs. Importantly, NNMT inhibitors (NNMTi) have been shown to reduce complement factor secretion by CAFs, decrease MDSC recruitment, and restore CD8^+^ T cell function, indicating that NNMTi can overcome CAF-mediated immunosuppression and enhance ICI response [25].

**Fig. 5:**
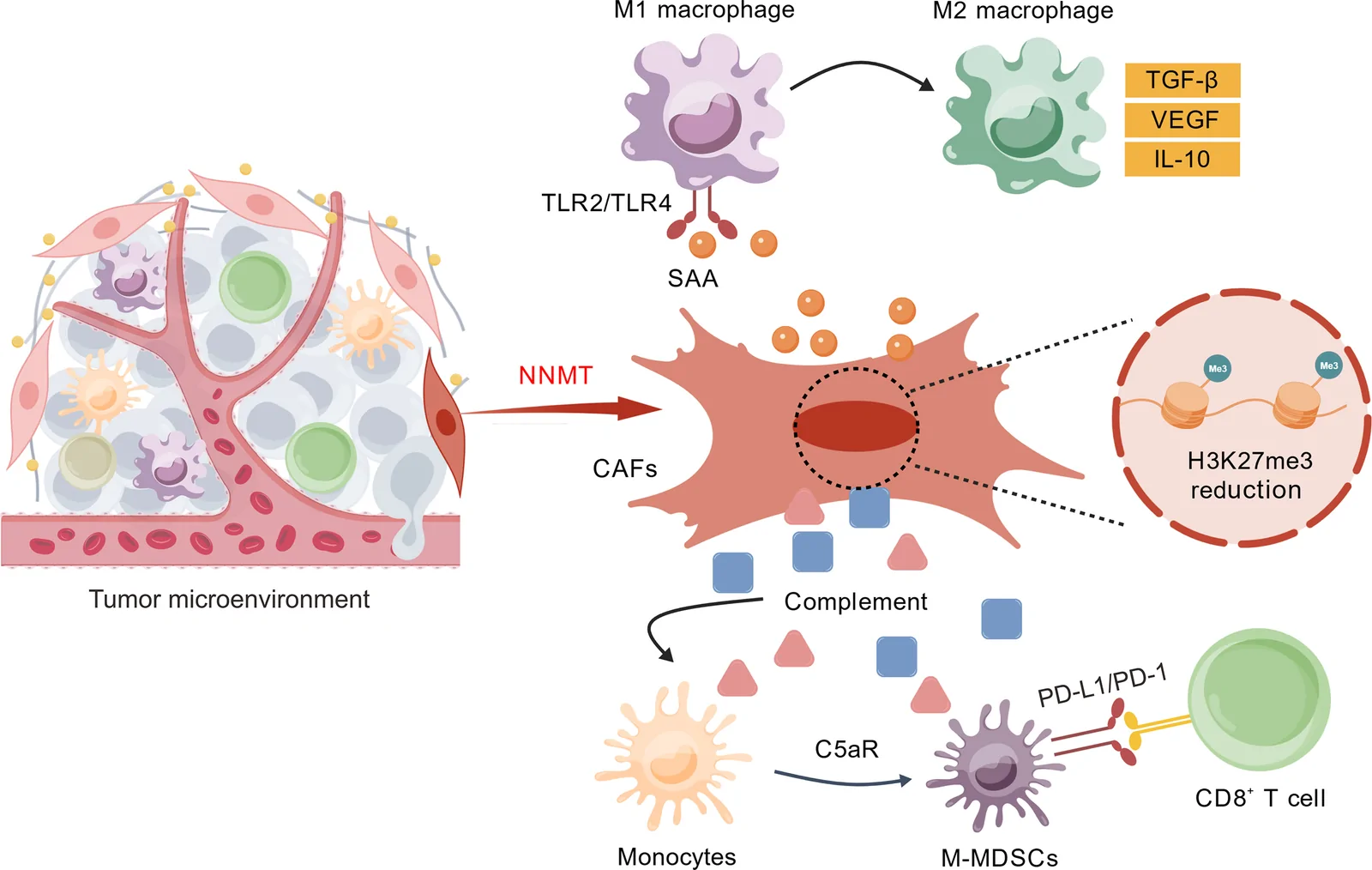
Cancer-associated fibroblasts (CAFs) with high nicotinamide N-methyltransferase (NNMT) drive immunosuppressive microenvironment and immunotherapy resistance. TLR2: Toll-like receptor 2; TGF-β: transforming growth factor beta; VEGF: vascular endothelial growth factor; IL-10: interleukin 10; SAA: serum amyloid A; H3K27me3: histone H3 lysine 27 trimethylation; C5aR: complement component 5a receptor; M-MDSCs: myeloid-derived suppressor cells; PD-L1/PD-1: programmed death-ligand 1/programmed death-1; CD8⁺: cluster of differentiation 8.

Given that NNMT participates in NAD^+^ metabolism by competing with nicotinamide phosphoribosyltransferase (NAMPT) for their common substrate NAM [84], it is plausible that NNMT may indirectly influence immune checkpoint regulation and contribute to immune resistance. Lv et al. [85] previously reported that NAMPT regulates PD-L1 expression in tumor cells via the interferon-gamma (IFN-γ)/signal transducer and activator of transcription 1 (STAT1) signaling pathway, facilitating immune escape. In line with this, clinical evidence indicates that upregulated NNMT in CAFs correlates with resistance to ICI treatment in multiple cancer types, such as urothelial bladder carcinoma (UBC) [30]. NNMT-high CAFs promote the expression of serum amyloid A (SAA) proteins within the tumor microenvironment. This enhances macrophage recruitment and polarizes them toward a tumor-promoting phenotype, facilitating immune evasion and contributing to poor ICI response [30]. Moreover, SAA proteins secreted by CAFs may also modulate other immunosuppressive populations such as MDSCs [86], further reinforcing an immune-tolerant microenvironment.

Additionally, MNAM, the primary metabolite generated via NNMT-mediated methylation of nicotinamide, exerts multifaceted roles in immune regulation. Jiang et al. [84] showed that CAFs with high NNMT expression elevate MNAM levels, which impairs CD8^+^ T cell function, promotes immunosuppression, and drives tumor progression and immunotherapy resistance. In high-grade serous ovarian cancer (HGSOC), MNAM induces tumor necrosis factor-alpha (TNF-α) secretion by T cells, establishing a pro-tumor inflammatory feedback loop. Inhibition of MNAM synthesis or export restores T cell-mediated antitumor immunity, highlighting its potential as an immunotherapeutic target [87]. Therefore, targeting NNMT-high CAFs represents a promising therapeutic strategy for overcoming ICI resistance.

#### 2.3.2 Potential involvement of NNMT in resistance to adoptive cell therapies

To date, there is no direct experimental evidence demonstrating that NNMT contributes to resistance against adoptive cell therapies such as CAR-T therapy (Table 1). However, this area remains largely unexplored, and future investigations are warranted to determine whether NNMT may influence the antitumor activity of adoptively transferred immune cells. Potential mechanisms could involve NNMT-mediated metabolic reprogramming or epigenetic modifications that alter the tumor microenvironment or immune cell functionality, thereby compromising therapeutic efficacy.

## 3 Self-amplifying NNMT feedback loops in cancer cells necessitate drug resistance to anticancer drugs

As described above, the high expression of NNMT is a key contributor to the development of drug resistance in cancer cells. Therefore, self-amplifying NNMT feedback loops in cancer cells necessitate drug resistance to anticancer drugs.

As illustrated in Fig. 6, by epigenetically activating ALDH3A1 and EGR1, elevated NNMT expression reduces the repressive histone marks H3K27me3 and H3K9me3 [29]. On one hand, EGR1 directly activates the transcription of *NNMT*, forming a self-sustaining regulatory loop (EGR1/NNMT/EGR1). A previous study demonstrated that NNMT promotes EGFR-TKI resistance in lung cancer by upregulating c-Myc expression [26]. Yao et al. [88] further revealed that c-Myc can directly bind to the *EGR1* promoter and transcriptionally activate *EGR1*. These findings suggest that c-Myc may contribute to TKI resistance in lung cancer by participating in the EGR1/NNMT/EGR1 feedback loop through transcriptional activation of *EGR1*. On the other hand, NNMT enhances ALDH3A1 expression via histone demethylation, facilitating increased lactate production. Elevated lactate levels then stimulate *NNMT* expression through H3K18 lactylation, establishing a second epigenetically mediated feedback loop: NNMT/ALDH3A1/lactate/NNMT [29]. ALDH3A1 also promotes glycolysis via activation of LDHA, thereby accelerating lactate secretion and supporting rapid cell proliferation, an axis known to contribute to both tumorigenesis and drug resistance [[89], [90], [91]].

**Fig. 6:**
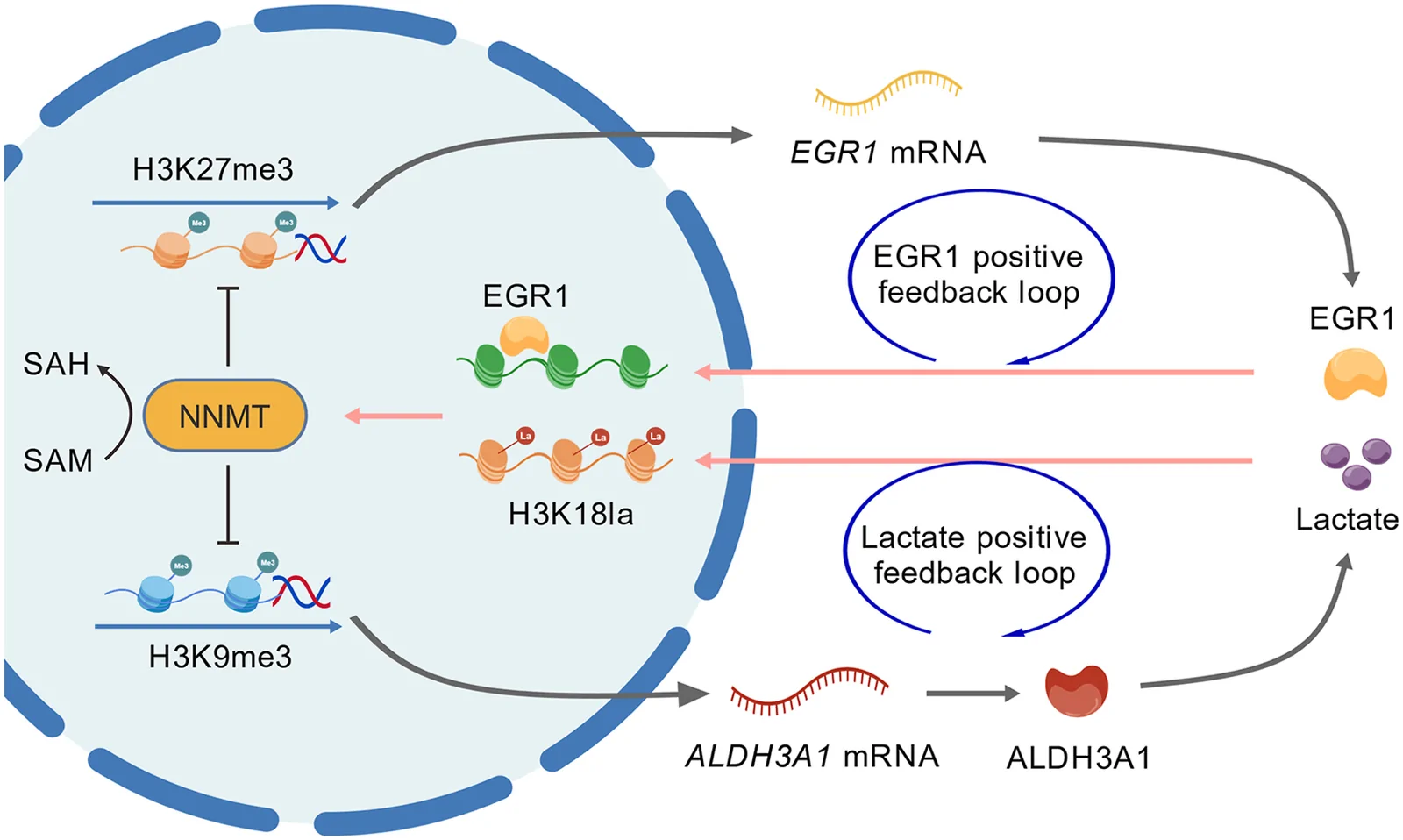
Nicotinamide N-methyltransferase (NNMT)-mediated self-activating feedback loop promotes drug resistance. H3K27me3: histone H3 lysine 27 trimethylation; SAH: S-adenosylhomocysteine; SAM: S-adenosylmethionine; H3K9me3: histone H3 lysine 9 trimethylation; *EGR1* mRNA: early growth response 1 messenger RNA; *ALDH3A1* mRNA: aldehyde dehydrogenase 3 family member A1 messenger RNA; H3K18la: histone H3 lysine 18 lacylation.

Additionally, the lactylation “writer” p300, which modifies histones at the *NNMT* promoter region, is upregulated in this context, further enhancing *NNMT* transcriptional activity. Zhang et al. [92] reported that ALDH3A1 may also stimulate oncogenic Akt signaling, which is closely linked to enhanced lactate production in NSCLC [93,94]. This suggests that Akt activation may operate in conjunction with ALDH3A1 to sustain lactate-driven positive feedback, thereby promoting TKI resistance.

Furthermore, beyond the established EGR1/NNMT/EGR1 and NNMT/ALDH3A1/NNMT dual positive feedback loops, NNMT can amplify its own expression and reinforce the drug-resistant phenotype through multiple indirect pathways. First, high NNMT activity depletes SAM, reducing methyl donor availability and subsequently relieving PR domain zinc finger protein 5 (PRDM5)-mediated H3K9me3 suppression at the *ALDH3A1* promoter [95]. This indirectly enhances ALDH3A1-lactate axis output, further promoting lactylation-dependent activation of *NNMT* expression. Second, the NNMT metabolite MNAM can be converted by aldehyde oxidase 1 (AOX1) into 2-pyridine and 4-pyridine derivatives [96]. These pyridine analogues inhibit PARP1 and attenuate ROS-dependent DNA damage signaling [97], creating a “low-ROS, pro-resistance” microenvironment that indirectly stabilizes HIF-1α and sustains upregulation of glycolytic genes such as *HK2* and *LDHA* [26], thereby recycling support for NNMT substrate supply. Third, SAM depletion suppresses DNMT activity, leading to DNA hypomethylation, epigenetic remodeling, downregulation of mitochondrial function-related genes, and impaired OXPHOS. This metabolic shift forces cancer cells to rely more heavily on glycolysis [43]. Thus, NNMT not only amplifies its own expression through direct positive feedback but also utilizes a multi-layer network of epigenetic, metabolic, and oxidative stress-related nodes to establish a self-reinforcing drug resistance circuitry.

As previously discussed, the Warburg effect in cancer cells maintains high levels of NAD^+^ and activates NNMT [50]. Once activated, NNMT enters a dual positive feedback loop mediated by lactate and EGR1, leading to self-activation of NNMT [29]. Consequently, cancer cells inevitably develop drug resistance through lactate-induced lactylation-mediated NNMT activation, a fundamental survival mechanism as long as malignant cells persist. Specifically, the Warburg effect in tumor cells induces tumor microenvironment remodeling through lactate accumulation, thereby activating NNMT and facilitating its autoactivation loop. Subsequently, NNMT enhances drug resistance by reducing cytotoxicity, weakening targeted inhibition, rendering monoclonal antibodies ineffective, and promoting immune evasion. Consequently, pharmacological inhibition of NNMT represents a viable strategy to both enhance the effectiveness of existing anticancer therapies and overcome resistance mechanisms.

## 4 Therapeutic targeting of NNMT to overcome drug resistance

### 4.1 Gene silencing strategies

Preclinical studies have demonstrated that gene-targeting interventions, such as short hairpin RNA (shRNA) or small interfering RNA (siRNA), can effectively suppress the growth of drug-resistant tumors and reverse resistant phenotypes. As summarized in Table 2 [[22], [23], [24],^28^,^29^,^36^], gene silencing has become a widely used experimental approach to investigate NNMT's role in tumor drug resistance. For example, genetic silencing of NNMT markedly increased cancer cell susceptibility to rapamycin treatment, whereas NNMT overexpression induced therapeutic resistance, as demonstrated by Yu et al. [22]. This study establishes *NNMT* as a critical regulator of drug response dynamics.

**Table 2:** Gene silencing studies targeting nicotinamide N-methyltransferase (NNMT) to overcome drug resistance.

Study focus	Cell line(s)	Key method	Key findings	Refs.
Rapamycin sensitivity	PANC-1	siRNA	si-NNMT cells showed approximately 50% lower viability at 10 nM (*P* < 0.01), and survival also significantly reduced at 50 and 100 nM.	[22]
5-FU sensitization (*in vitro/in vivo*)	TE1	siRNA	IC_50_ significantly decreased, reduced viability *in vitro*; smaller tumors and improved 5-FU response *in vivo*.	[23]
Resistance to ADM/PTX	MDA-MB-231	shRNA	NNMT knockdown led to a marked decrease in ADM and PTX IC_50_, indicating increased chemosensitivity.	[24]
5-FU sensitivity	AGS	shRNA	IC_50_ decreased from 21.37 to 12.22 μg/mL, and viability also reduced.	[28]
BRAF inhibitor resistance	Bics	siRNA	Genetic silencing and pharmacological inhibition of NNMT significantly reduced cell viability and colony formation ability in three resistant melanoma cell lines.	[36]
EGFR-TKI resistance	NSCLC	shRNA	Gefitinib reduced IC_50_ from 1.5 to 0.5 μM (PC9/GR), 2.0 to 0.8 μM (HCC827/GR); osimertinib reduced IC_50_ from 1.2 to 0.6 μM (PC9/OR) and 1.8 to 0.9 μM (HCC827/OR).	[29]

siRNA: small interfering RNA; shRNA: short hairpin RNA; si-NNMT: siRNA targeting NNMT; IC_50_: half maximal inhibitory concentration; ADM: doxorubicin; PTX: paclitaxel; 5-FU: 5-fluorouracil; BRAF: v-raf murine sarcoma viral oncogene homolog b; EGFR-TKI: epidermal growth factor receptor-tyrosine kinase inhibitor; NSCLC: non-small cell lung cancer.

Although NNMT silencing or knockout has shown promising effects *in vitro* and in animal models, the translation of these strategies into clinical applications remains challenging [15,98]. Barriers include delivery efficiency, off-target effects, and the complexity of tumor heterogeneity. Therefore, future research should focus on optimizing NNMT-targeting technologies, elucidating tumor-specific regulatory mechanisms, and validating safety and efficacy through well-designed clinical trials across diverse cancer types.

### 4.2 Small-molecule inhibitors

NNMT regulates drug sensitivity, treatment efficacy, and tumor evolution, making it an attractive therapeutic target against cancer drug resistance. As summarized in Table 3 [25,26,29,30,[36], [37], [38],[99], [100], [101], [102], [103]], across various preclinical tumor models, multiple small-molecule NNMTi have demonstrated efficacy in reversing acquired chemoresistance.

**Table 3:** Summary of small-molecule nicotinamide N-methyltransferase (NNMT) inhibitors.

Inhibitor name	Structure and compound type	Characteristics	IC_50_	Refs.
6-Methoxynicotinamide		High selectivity and favorable pharmacokinetics; exhibits slow turnover resulting in prolonged *in vivo* clearance	1.8 μM	[26,37,99]
5-AMQ		Highly selective; does not inhibit other SAM-dependent methyltransferases or key enzymes in NAD^+^ repair pathways	1.2 ± 0.1 μM	[29,30,36,100]
YD		Mechanism not fully elucidated	0.4 μM	[38,101,102]
Vanillin		Food-grade additive with low toxicity *in vitro* and *in vivo*	Approximately 2.5 mM (effective concentration at cellular level)	[103]
NCGC00685960		A highly efficient NNMT inhibitor with favorable oral bioavailability and specificity	<10 nM	[25]

5-AMQ: 5-amino-1-methylquinolinium; SAM: S-adenosylmethionine; NAD^+^: nicotinamide adenine dinucleotide; YD: yuanhuadine; IC_50_: half maximal inhibitory concentration.

Bach et al. [38] found that Yuanhuadine (YD), a natural compound, restored the sensitivity of NSCLC cells to 5-FU by inhibiting NNMT enzymatic activity. Co-treatment with YD and an EGFR-TKI significantly enhanced antitumor effects. Further mechanistic studies by Hong et al. [104] demonstrated that YD exerted antiproliferative activity via cell cycle arrest and suppression of the Akt/mammalian target of rapamycin (mTOR) and EGFR signaling pathways. Notably, the combination of YD and gefitinib exhibited synergistic cytotoxicity in NSCLC cells. Vanillin, a compound derived from the Chinese herb vanilla, has been demonstrated to directly inhibit NNMT enzymatic activity and downregulate NNMT expression in CRC cells [103]. *In vivo* models further confirmed that vanillin (100 mg/kg, administered intraperitoneally every other day) in combination with 5-FU effectively reduced tumor volume, highlighting its potential as a natural monomeric compound targeting NNMT for therapeutic applications [103].

In a separate study, Wang et al. [26] showed that the NNMTi, 6-methoxynicotinamide, when used in combination with EGFR-TKIs, effectively suppressed the growth of resistant NSCLC cell lines. Similarly, Dai et al. [29] found that 5-amino-1-methylquinolinium (5-AMQ), a selective NNMTi, significantly reduced the proliferation of EGFR-TKI-resistant cancer cells and sensitized them to targeted therapy.

Importantly, the therapeutic utility of NNMTi extends beyond targeting intrinsic resistance. Yang et al. [30] revealed that 5-AMQ, in combination with anti-PD-L1 immunotherapy, markedly suppressed tumor growth, enhanced CD8^+^ T cell cytotoxicity, and reduced infiltration of immunosuppressive M2 macrophages. These findings suggest that NNMT inhibition may also synergize with immune checkpoint blockade, offering a dual approach to overcome both metabolic and immune-mediated resistance. In the study by Ghezzi et al. [36], it was observed that treatment of BiR with 5-AMQ led to a significant reduction in cell proliferation and an increased sensitivity to BRAF inhibitors. Similarly, silencing the NNMT gene using siRNA technology also yielded comparable outcomes. Hou et al. [37] confirmed that NNMT expression significantly increased following AZD9291 treatment, particularly in drug-resistant cells. Overexpression of NNMT was found to diminish the therapeutic efficacy, whereas the NNMTi, 6-Methoxynicotinamide, enhanced the treatment response.

Heide et al. [25] conducted a high-throughput screening of 152,778 small molecule compounds, identifying 361 highly active lead structures. From these, they developed a highly effective NNMTi (NCGC00685960). This inhibitor significantly suppresses CAF-driven MDSC recruitment, markedly reduces tumor growth and metastasis *in vivo*, and enhances the therapeutic efficacy of immune checkpoint blockade (ICB) [25]. Furthermore, NNMTi demonstrates favorable oral bioavailability and high specificity, highlighting its substantial potential for clinical application.

### 4.3 Intervention strategies targeting positive feedback loops

Dai et al. [29] elucidated a molecular mechanism in which NNMT drives drug resistance through dual positive feedback loops mediated by lactate and EGR1. Theoretically, disrupting any critical node within these feedback circuits can interrupt NNMT's self-activation, thereby reducing resistance.

One potential approach is to inhibit EGR1 expression or activity using siRNA or small-molecule inhibitors. For instance, Liu et al. [67] reported that EGR1 is upregulated in ibrutinib-resistant B-cell lymphoma, where it promotes OXPHOS metabolic reprogramming and supports resistant cell proliferation. Pharmacological inhibition of OXPHOS with agents such as IM156 and metformin effectively suppressed the growth of resistant cells, representing a novel therapeutic strategy against resistance. However, direct targeting of EGR1 may be complicated by its involvement in diverse regulatory networks and the risk of off-target effects [105,106].

Another intervention point involves suppressing ALDH3A1 activity or expression. Narendra et al. [107] utilized machine learning to identify bazedoxifene and raloxifene as selective inhibitors of aldehyde dehydrogenase 1 family member A1 (ALDH1A1). Enzymatic assays showed that bazedoxifene and raloxifene inhibit ALDH1A1 with half-maximal inhibitory concentration (IC_50_) values of 4.41 and 2.35 μM, respectively, while exhibiting minimal inhibition of ALDH3A1 and ALDH2. Moreover, combined administration of these agents enhanced cyclophosphamide sensitivity *in vitro*, suggesting their potential as adjuvants to improve therapeutic efficacy and reduce toxicity.

Collectively, these findings indicate that targeting key components of the NNMT-driven feedback loops, either upstream transcription factors like EGR1 or downstream metabolic enzymes such as ALDH3A1, offers promising avenues to overcome tumor drug resistance. Further development of specific inhibitors and combination regimens warrants exploration in preclinical and clinical settings.

## 5 Implications for future research

### 5.1 Interaction between lactate and NNMT: a new opportunity in metabolic reprogramming and epigenetic regulation

The tumor-specific metabolic shift toward excessive lactate generation via the Warburg effect fundamentally reprograms the local microenvironment. This metabolic shift not only meets cellular energy demands but also alters intracellular metabolic networks [47,48]. Accumulated lactate raises intracellular NAD^+^ levels, resulting in excess NAM. NAM is methylated by NNMT to MNAM, which, while depleting NAM pools, accelerates its turnover and thereby supports tumor cell survival [17]. From an epigenetic perspective, NNMT-mediated reduction of repressive histone marks (H3K27me3/H3K9me3) activates ALDH3A1 to boost lactate production, which reciprocally enhances *NNMT* expression through H3K18 lactylation, forming a metabolic feedforward cycle [29].

This interplay reveals a close link between metabolic reprogramming and epigenetic regulation, providing a theoretical basis for novel cancer therapies. Targeting NNMT or its metabolic products to disrupt the lactate-mediated positive feedback loop holds potential to inhibit tumor progression. Furthermore, elucidating the role of this interaction within the tumor microenvironment will shed light on tumor-immune system dynamics. Overall, the lactate-NNMT axis represents a promising frontier at the crossroads of metabolism and epigenetics with potential breakthroughs for cancer treatment.

### 5.2 Potential existence of alternative NNMT-mediated positive feedback loops

Bach et al. [38] reported a positive feedback loop in NSCLC involving PTEN-controlled PI3K/Akt signaling and microRNA-449a (*miR-449a*)-mediated NNMT expression, which contributes to increased proliferation and reduced gefitinib sensitivity. Specifically, elevated NNMT expression enhances methylation of the *PTEN* promoter, suppressing *PTEN* expression [38]. PTEN downregulation activates PI3K/Akt signaling, which further upregulates NNMT via *miR-449a*, thus reinforcing the loop [38]. However, theoretically, increased NNMT activity decreases intracellular SAM levels, reducing global methylation capacity. Therefore, *PTEN* promoter methylation would be expected to decrease, promoting *PTEN* expression rather than repressing it. This discrepancy suggests the involvement of additional, yet unidentified, indirect mechanisms by which NNMT overexpression leads to hypermethylation of the *PTEN* promoter, warranting further experimental investigation.

### 5.3 The exercise-lactate-lactylation axis and NNMT: a hypothesis to be tested

Exercise exhibits multiple beneficial effects in cancer prevention and treatment. Studies have demonstrated that beyond lowering cancer incidence [108], exercise can enhance the efficacy of approved anticancer therapies and alleviate symptoms and side effects such as depression, cognitive impairment, cachexia, and fatigue [[109], [110], [111], [112]]. Mechanistically, lactate generated during and after intense exercise can directly influence the epigenetic landscape. For example, Han et al. [113] found that exercise increases brain lactate levels and H3K18la, promoting microglial polarization from pro-inflammatory to anti-inflammatory/repair phenotypes, thereby ameliorating neuroinflammation and cognitive deficits in Alzheimer's disease mouse models. Sodium lactate administration mimicked these effects, underscoring the role of lactylation in regulating microglial function and cognition. Xu et al. [114] showed that exercise training reduces cardiac lactate levels and H3K18la, suppressing YTH m^6^A RNA binding protein 2 (YTHDF2) expression and mitigating myocardial ischemia-reperfusion injury. Given that histone lactylation enhances *NNMT* expression, it is plausible that exercise-induced lactylation in skeletal muscle elevates *NNMT* expression.

MNAM, as the catalytic product of NNMT, acts as its natural inhibitor through feedback regulation [115]. This raises a critical scientific question: could elevated circulating MNAM levels resulting from increased muscle *NNMT* expression systemically inhibit NNMT activity in tumor tissues through such feedback mechanisms? If validated, this would establish a novel metabolic axis termed “exercise-muscle lactylation-NNMT-MNAM-tumor metabolic suppression,” providing a new perspective for understanding exercise-assisted antitumor effects.

### 5.4 Does NNMT regulate NSCLC cells through additional metabolic pathways?

NNMT has been shown to regulate glycolysis-related metabolic pathways, affecting cancer cell proliferation and migration partly via epigenetic modulation of glycolytic enzyme gene methylation and expression [23]. This suggests that NNMT may serve not only as a regulator of individual metabolic pathways but also as a hub linking multiple metabolic circuits. However, whether NNMT influences key enzymes in such pathways via epigenetic mechanisms remains unclear. Future studies should investigate whether NNMT regulates glutamine metabolism enzymes through methylation modifications and assess its functional consequences in NSCLC cells. Systematic elucidation of NNMT's regulatory roles across diverse metabolic pathways could expand therapeutic targets in cancer metabolism and reveal molecular bases for functional heterogeneity among tumor types.

## 6 Conclusion

This review establishes NNMT as a pivotal metabolic-epigenetic driver of cancer drug resistance, mediated through diverse mechanisms and self-amplifying feedback loops. While NNMT-targeting strategies show preclinical promise, overcoming tumor heterogeneity and biomarker gaps remains critical for clinical translation. Future research must prioritize decoding NNMT-metabolite crosstalk (e.g., lactate), identifying novel feedback circuits, and designing NNMT-inclusive combination regimens. Elucidating these axes will accelerate the development of effective resistance-breaking therapies to improve patient survival.

## CRediT authorship contribution statement

**Shuang-Zhou Bi:** Writing – original draft. **Xiang-Li Ni:** Conceptualization. **Wei-Dong Sun:** Conceptualization. **Chen-Ying Zhang:** Conceptualization. **An Liu:** Conceptualization. **Shi-Yan Lai:** Conceptualization. **Jiang-Hua Li:** Writing – review & editing, Funding acquisition.

## Declaration of competing interest

The authors declare that they have no known competing financial interests or personal relationships that could have appeared to influence the work reported in this paper.
